# Physio-fEedback and Exercise pRogram (PEER) for Shifting Maladaptive Fall Risk Appraisal

**DOI:** 10.1093/geroni/igaa057.3092

**Published:** 2020-12-16

**Authors:** Ladda Thiamwong

**Affiliations:** College of Nursing, University of Central Florida, Orlando, Florida, United States

## Abstract

We aimed to examine the effectiveness of Physio-feEdback and Exercise pRogram (PEER) for shifting maladaptive to adaptive fall risk appraisal and determine the feedback and acceptability of the program. Forty-one older adults were assigned to either PEER intervention or attention control (AC) group. The 8-week PEER intervention consists of a visual physio-feedback, cognitive reframing, and combined group and home-based exercise led by a trained peer coach. The AC group read fall prevention brochures and continued their normal activities. BTrackS Balance Test and Fall Efficacy Scale were measured from pre- to post-intervention. About 11% of participants in the PEER group had positive shifting but none in AC group. Up to 32% of the participants in AC had negative shifting while 5.3% in the PEER group. PEER intervention facilitates a shift from maladaptive to adaptive fall risk appraisal. PEER group reported significant decreases in fall risk and high acceptability of the program.

